# What Do You Think About Your Company’s Leaks? A Survey on End-Users Perception Toward Data Leakage Mechanisms

**DOI:** 10.3389/fdata.2020.568257

**Published:** 2020-10-30

**Authors:** Yoann Bertrand, Karima Boudaoud, Michel Riveill

**Affiliations:** Université Côte d’Azur, CNRS, I3S, Sophia Antipolis, France

**Keywords:** security, data leakage, access control, transmission control, security policies, usability, online survey, end-users perception

## Abstract

Data leakage can lead to severe issues for a company, including financial loss, damage of goodwill, reputation, lawsuits and loss of future sales. To prevent these problems, a company can use other mechanisms on top of traditional Access Control. These mechanisms include for instance Data Leak Prevention or Information Rights Management and can be referred as Transmission Control. However, such solutions can lack usability and can be intrusive for end-users employees. To have a better understanding of the perception and usage of such mechanisms within business infrastructures, we have conducted in this article an online survey on 150 employees. These employees come from different companies of different sizes and sectors of activity. The results show that whatever the size of the company or its sector of activity, security mechanisms such as access control and transmission control can be considered as quite intrusive and blocking for employees. Moreover, our survey also shows interesting results regarding more acceptable and user-friendly anti-data leakage mechanisms that could be used within companies.

## Introduction

While doing business, a company creates, exchanges and saves meaningful data. These meaningful data are valuable for the company wealth, good functioning and reliability. Thus, a company has to protect these information. To do so, a company can use Access Control (AC), which is a set of techniques that restrict the access to resources to specific and authorized users by defining “who can access what?” However, due to the growth of networks and connected computers, a security issue, called data leakage, has arisen.

Data leakage has been defined as the unauthorized distribution of confidential or sensitive data (by sensitive, we include information that poses a risk to a company if discovered by a competitor or the general public.) to an unauthorized entity (Shabtai et al., 2012). For this reason, data leakage can create various problems for a company, such as financial loss, damage of goodwill and reputation, lawsuits, loss of future sales or exposure of intellectual properties (Cisco Systems, 2009). The cause of leakage can either be external (i.e., from outsiders) or internal (i.e., from employees, collaborators, etc.). Moreover, data leakage can be:

- *Intentional*: which means done with full knowledge of the facts (i.e., I know that I do not have the right to do it, for example sending a document, or I bypass on purpose the security mechanism). Intentional data leakage can be done by a spy, hacker or a malicious employee motivated by grudge or revenge;
- *Unintentional*: which means done without full knowledge of the facts and without intent to cause harm. This kind of data leak can be due to human errors, lack of awareness, clumsiness, etc.

In this paper, we focus on unintentional data leakage.

To avoid data leakage, a company can use other solutions besides AC, such as Data Leak Prevention (DLP) and Information Rights Management (IRM). However, these mechanisms can be complex to apprehend (i.e., not easy to understand and use) for end-users. For example:

- Some end-users may have difficulties to understand why they need to apply specific security mechanisms and follow security rules. Even if most of users understand that security is a fundamental need, the security restrictions can be seen by employees as a lack of confidence from the employer or a depreciation of their skill and professionalism.
- Some end-users may struggle with security mechanisms when doing their work, which can be frustrating and very time-consuming and push some of them to bypass security rules.
- Some employees, in the case of small companies, may need to understand the technical basics of the security mechanisms (such as security of data at rest, security of data in-use and security of data in-motion, etc.) even if they do not have the knowledge.

Having a security mechanism that is difficult to apprehend does not help to adopt it. Generally, most of people/end-users “give up” when a security mechanism become complex to understand and/or use. Thus, what is needed is an anti-data leakage solution that is user-friendly, usable, acceptable and non-intrusive for the employees of a company. In order to provide such solution, we present in this article a survey we have conducted among employees of several companies. This survey serves the following purposes:

- Gather information on the employees' position, computer skills and sector of activity.
- Gain insight on the employee’s knowledge and perception toward access control and data leakage.
- Determine the mechanisms the employees would prefer to prevent them from performing unintentional data leakage.

Regarding the activity sector, we target all kind of domains:

- Information and Communication Technology
- Health
- Energy
- Defence
- Consulting
- Scientific, Research, and Development
- Industrial (mechanic, cars, metallurgy, etc.)
- Construction
- Consumer goods, Transport, Hotel, Food, Catering
- Insurance/Banking/Financial institution
- Housing
- Public administration, academic

The rest of the article is structured as follows: “Related Works” section gives an overview about existing works on access control, data leak prevention, information right management and surveys on security and usability. “Online Survey**”** details our survey. Sections “Feedbacks on Context, Participants’ Positions and Skills,” “Feedbacks on Awareness, Knowledge and Perception Towards Security Policies and Data Leakage” and “Feedbacks Towards Data Leak Prevention Mechanisms” present the results we have obtained. “Discussion” section discusses the most interesting correlations we have identified. Finally, “Conclusion and Future Works” section concludes the article and give insights on future works.

## Related Works

Many existing works have focused on designing and developing AC, DLP, and IRM mechanisms to prevent data leakage. All these mechanisms have advantages and disadvantages from a technical point of view. However, even if these mechanisms are powerful, unintentional data leakage happens in all kinds of activity sector (see Table 1). This is mainly due to human factors (Kirlappos et al., 2014; Alotaibi et al., 2016; Beautement et al., 2016; Wan Basri and Maryati, 2018) as these mechanisms are generally not user-centric (Workman et al., 2008; Beautement et al., 2016) and have been designed without considering the point of view of the end-user. Several studies have been conducted to analyze human factors regarding the use of security mechanisms and security policies (Pahnila et al., 2007; Workman et al., 2008; Rhee et al., 2009; Kirlappos et al., 2014; Beautement et al., 2016). However, to the best of our knowledge, none of these studies have focused on the end-users perception regarding data leakage within companies.

**TABLE 1:** Some example of real cases of data leakage.

Date	Organization	Information
2007	U.S. Nuclear Laboratory	An employee transmitted confidential information on US atomic weapons by email via non-secured networks to members of the board of los Alamos National security
2008	Norway Government	The tax agency mistakenly sent CDs containing confidential information about nearly 4 millions (i.e., 85%) of Norwegian adults to nine major media groups
2011	Sogeti	A file containing the personal information and evaluations of 298 employees was unintentionally sent by email to these employees. Among personal information such as salaries and raise the file included commentaries on employees performances
2016	Google	A company’s staff benefits vendor mistakenly sent an email containing employee’s sensitive information to the wrong recipient
2016	Australian Government	An administrative error from the prime minister’s department revealed a mailing list of 800 addresses that were supposed to be confidential
2016	Federal Deposit Insurance Corporation	Former employee accidentally made a copy of 44.000 customers on a USB drive

In this section, we first give an overview about the main works on Access Control, Data Leak Prevention and Information Right Management. However, we will not compare these mechanisms, as this is not the goal of this work. Then, we outline the existing surveys conducted regarding usability and security.

### Access Control

Access Control (AC) aims at restricting access to resources. Traditionally, Access Control can be divided in two categories: Discretionary Access Control (DAC) (Lampson, 1974; Saltzer and Schroeder, 1975; Harrison et al., 1976) and Mandatory Access Control (MAC) (Bell and LaPadula, 1973; Biba, 1977). In DAC models, users can set, modify or share the access control of their resources. Most modern operating systems such as Windows, GNU/Linux and Mac OS are based on DAC models. On the contrary, MAC refers to a family of models where owners do not have to choose the rights over their resources. In this type of access control, the system assigns security labels or classifications to resources (for instance “classified,” “secret,” or “top secret”) and allows access to subjects or applications depending on their level of clearance. Over the last decades, several models have been proposed to cover the problem of Access Control. These models propose to take into account various notions, including roles (RBAC) (Sandhu, 1996), attributes (ABAC) (Hu, 2013), context (Corrad, 2004), history (Banerjee and Naumann, 2004), risk (Kandala et al., 2011), authorization (Karp et al., 2010) or trust (Kagal et al., 2001).

### Data Leak Prevention

In Shabtai et al., 2012, a DLP has been described as a “system that monitors and enforces policies on fingerprinted data that are at rest (i.e., in storage), in-motion (i.e., across a network) or in-use (i.e., during an operation) on public or private computer/network.” DLPs are usually based on policies. These policies can help security experts and administrators to prevent data leakage by defining rules such as “send an email when user U1 sends document X to user U2.” Since 2006, several larger vendors have bought smaller companies specialized in data security.^b^ Thanks to these buyouts, DLPs technologies have started to arise since 2008, proposing scalable and business oriented solutions. Nowadays, the biggest vendors are Websense,^c^ Trend Micro,^d^ RSA,^e^ Symantec^f^ and Palisade Systems.^g^

From the academic point of view, researchers have focused on several problems, including emails leakage protection (Zilberman et al., 2011), network and Web based protection (Caputo et al., 2009) and misuse detection in database (Harel et al., 2010; Harel et al., 2012). Moreover, solutions have been proposed to improve detection methods by using machine learning (Gafny et al., 2010; Mathew et al., 2010; Li et al., 2015). Closer to industrial preoccupations (Alawneh and Abbadi, 2008), have proposed a framework to protect the data shared between collaborative organizations. Finally, some works have been proposed to tackle sensitive data (Chae et al., 2015) or confidentiality (di Vimercati, 2011).

### Information Rights Management

IRM is a subset of Digital Rights Management (DRM). A DRM is a mechanism that aims at preventing unauthorized redistribution of a digital media (e.g., document, music, video) and restricts the ways consumers can use this content (copy, distribution to others, etc.). DRM solutions have been developed in response to the increase of online piracy (i.e., redistribution of copyrighted information over the Internet thanks to peer-to-peer networks). Within companies, IRM [Other names, such as Enterprise DRM, can also be found in the research field] can be used. IRM refers to Rights Management technology specifically designed for enterprise documents. Thus, IRM aims at protecting sensitive information, such as patents, employees personal information or financial data.

The main vendors in the domain are Seclore,^h^ Microsoft,^i^ Covertix,^j^ and EMC.^k^ From the academic point of view, traditional DRM have been discussed in many papers (Rosenblatt et al., 2001; Subramanya and Yi, 2006; Van Tassel, 2006). Different IRM solutions have also been proposed and compared (van Beek, 2007), especially to tackle problems such as insiders leak (Yu and Chiueh, 2004), usage tracking (Yang et al., 2013) and storage efficiency issues (Soliman et al., 2015).

### Surveys on Security and Usability

Many surveys on security and usability have been proposed over the years. For instance, specific types of users, such as administrators or security experts have been targeted by surveys such as CryptzoneSurvey.^l^ In this survey, administrators have been asked to give insights on the usage of network Access Control technologies and best practices. In SANS survey,^m^ security experts have been solicited to have insights on end-users security behavior. Closely to end-users themselves, security and usability have been studied in many ways, covering fields such as privacy (Kumaraguru and Cranor, 2005) and behavior (Beautement et al., 2016). Moreover, studies have been conducted to determine the perception of security properties such as confidentiality (Bai et al., 2016; Ruoti et al., 2016) and authentication (Stobert and Biddle, 2014) while other works have proposed mechanisms such as recommendation systems (Liu et al., 2016) and indicators (Felt et al., 2016) to help the end-users.

Finally, other works have been proposed in the DLP field to take into account usability. For instance (Ko et al., 2014), have proposed a user-centric mantrap-inspired DLP solution, implemented in Debian Linux to inform end-users about potential data leak and allow them to fully decide sending or not the data. In addition, DLP vendors such as Clearswift^n^ and Teramind^o^ propose adaptive dashboards and reporting in order to make their products more usable and user-friendly. However, to the best of our knowledge, no survey has been proposed to specifically target the end-users perception toward data leakage within companies. Thus, we aim at gathering information on this topic by proposing an online survey. This survey is described in the next section.

## Online Survey

In this Section, we present the online survey that we have designed to collect data from a broad audience of employees having different profiles (in terms of position and computer skill) and working in different companies (in terms of size and activity sector). The ultimate goal was to gather information on employees and their awareness, perception and knowledge regarding security policies and data leakage, within their working environment, to investigate three research questions:

- What is the knowledge and perception of employees regarding security policies, more specifically Access Control (“who can access what?”) and Transmission Control (“Who can send what to whom?”), and data leakage.
- Are the employees aware of what they can or cannot do?
- What are the mechanisms they would prefer to avoid unintentional data leakage?

Therefore, we have defined the most pertinent questions to collect useful answers for these research questions and have feedbacks of the participants on:

- Context (size of the company and activity sector), position and skill;
- Awareness, knowledge and perception toward security policies and data leakage;
- Data leak prevention mechanisms.

The survey included closed-ended multiple-choice questions, open-ended questions, and rating questions using a Likert scale. It was implemented using Google Form and was composed of 16 questions. The survey has been proposed in English^p^ and French^q^ and has been online for 10 months. Concerning the answers, we have gathered 150 results by proposing our survey through social media (LinkedIn, Twitter), personal contacts list and personal Website. Table 2 gives information on the questions and the type of answers. In the next subsections, we present the results we have obtained.

**TABLE 2:** Questions and types of answers of our online survey.

ID	Questions	Types of answer
1	What is the sector of your company?	Radio button
	• Industrial (mechanic, cars, metallurgy, etc.)	
	• Construction	
	• Consumer goods, transport, hotel, food, catering	
	• Information and communication	
	• Insurance/banking/financial institution	
	• Housing	
	• Scientific, research, and development	
	• Public administration, academic	
	• Health	
	• Energy	
	• Defense	
	• Consulting	
	• Other	
2	What is the size of your company?	Radio button
	• Between 0 and 19 employees	
	• Between 20 and 249 employees	
	• Between 250 and 5,000 employees	
	• More than 5,000 employees	
3	What is your position?	Short text area
4	How would you rate your computer skills?	Likert scale (1–7)
5	In your work, do you have to manage sensitive data?	Radio button
6	If so, do you often manipulate such data?	Radio button
	• Rarely (“it is never common in my work to manipulate sensitive data.”)	
	• Often (“from time to time, I have to manipulate sensitive data.”)	
	• Frequently (“A significant part of my work is to manipulate sensitive data.”)	
7	In your company, are you aware of the security policies that concern you?	Radio button
	By security policies, we mean policies of access control (“who can access what?”) and transmission control (“who can send what to whom?”)	
	• Yes, I know what I can/cannot do	
	• No, I know that they exist, but I do not know what I can do/cannot do	
	• No, and I do not know if they exist	
8	If you are aware of such policies, have you ever done anything to bypass them?	Radio button
	Example: Send a document to someone who had no access to this resource	
	• Yes, and I was aware that I was bypassing security	
	• Yes, but I was not aware that I was bypassing security	
	• No, never	
9	Do you think that not being aware of such policies is an obstacle for your work?	Likert scale (1–5)
10	According to you, data leakage can be	Radio button
	• A problem for the company (financial loss, image, etc.)	
	• A problem for the employee (official warning, dismissal, etc.)	
	• A problem for both	
	• A problem for neither of them	
11	Are you aware of security mechanisms implemented within your company?	Radio button
12	Do you think that these mechanisms are an obstacle to your work?	Likert scale (1–5)
13	Do you think that these mechanisms are intrusive for employees?	Likert scale (1–5)
14	According to you, what are the most efficient mechanisms to avoid data leakage?	Checkboxes
	• Notify users that an action is going to cause a data leakage and let her/him choose (e.g., popup messages)	
	• Notify users that an action is going to cause a data leakage and prevent this action	
	• Send an email to the administrator/manager to notify her/him and automatically prevent the action	
	• Send an email to the administrator/manager in order for her/him to choose if the action can be performed or not	
	• Automatically deactivate actions that can cause data leakage (for instance, automatically deactivate the “send” button when a confidential attached document is put within an email)	
	• Prevent the action without notifying the user	
	• Other form of mechanisms (you can share ideas at the end of the form)	
15	In the end, you would prefer a mechanism that let you decide, but might let you perform data leakage or a mechanism that prevent you from unintentional data leakage, but does not let you decide	Radio button
16	If you have any ideas of mechanisms, some remarks or comments.	Text area

## Feedbacks on Context, Participants’ Positions and Skills

In this subsection, we first present the results we have obtained concerning the context (i.e., size of the companies and sectors of activity). Then, we present the results on the participants’ positions and computer’s skills.

### Sectors of Activity

**FIGURE 1:**
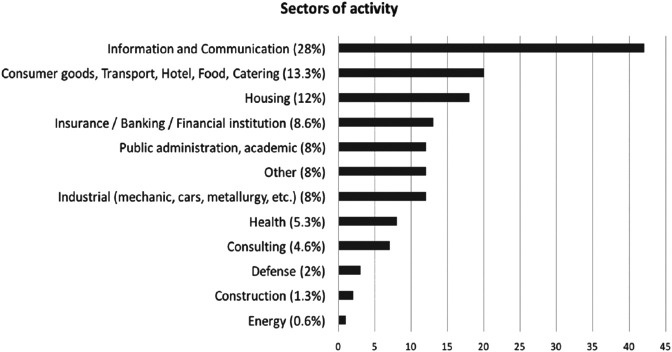
Distribution of the different sectors of activity.

The question 1 (*What is the sector of your company?*) of the survey is related to the sector the participants are working in Figure 1 shows various results due to the fact that different mediums have been used to share the survey. Thus, various sectors are represented, such as IT (28%), business/transport/hotel/food/catering (13.3%), housing (12%) or Insurance/banking/financial institution (8.6%). Other fields, such as defense (2%) construction (1.3%) or energy (0.6%) are anecdotal.

### Size and Types of Companies

**FIGURE 2:**
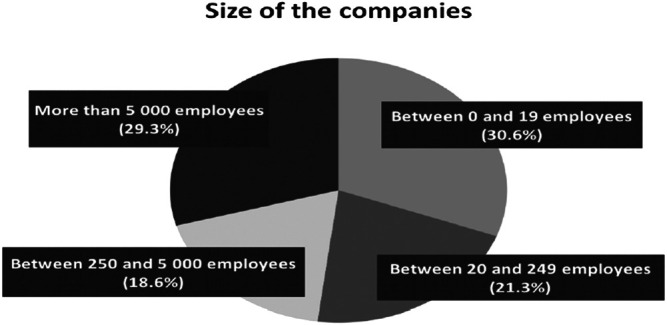
Distribution of the size of the companies.

Thanks to the question 2 (*What is the size of your company?*)*,* we have underlined that all sizes of companies are also represented. Indeed, results in Figure 2 show that roughly 52% (30.6 + 21.3) of the participants work in companies that employ less than 250 employees. Moreover, results show that big companies (i.e., strictly more than 5,000 employees) are also well represented (29.3%).

### Position of the Participants

Thanks to the question 3 (*What is your position?*), the survey shows various results regarding the position of the participants. Indeed, Table 3 shows a snippet of the results we have obtained. As one can see, various profiles can be underlined, including technical positions such as IT engineers, administrators, developers and security experts. Moreover, other profiles can be highlighted, such as accountant, bankers, Ph.D. students/researchers and employees from human resources. Finally, other types of participants have also answered the survey, including librarians, CEOs, professional photographs and building architects.

**TABLE 3:** Snippet of some of our participants’ positions.

Position	Number of participants fitting a position	Sector of activity
Accountant	6	Insurance/banking/financial institution
Solution architect	1	Information and communication
Community manager	1	Information and communication
Head of vessel finance team	2	Consumer goods, transport, hotel, food, catering
Librarian	3	Public administration, academic
Senior risk advisor	1	Insurance/banking/financial institution
Entrepreneur	3	Other
Operation executive	1	Consulting
CEO	1	Housing
Investigator	1	Defense
Broker	2	Insurance/banking/financial institution
SEO specialist	1	Information and communication
Web designer	1	Information and communication
Architect	5	Energy
Business analyst	1	Insurance/banking/financial institution
IT security expert	1	Industry
International sales and Marketing manager	1	Consumer goods, transport, hotel, food, catering

**TABLE 4:** The average perceived skill for each size of company.

Size of the company	Average perceived skill
Micro-enterprise	4.54
SME	4.65
MidCaps company	5.61
Large company	5.56

### Computer Skills

**FIGURE 3:**
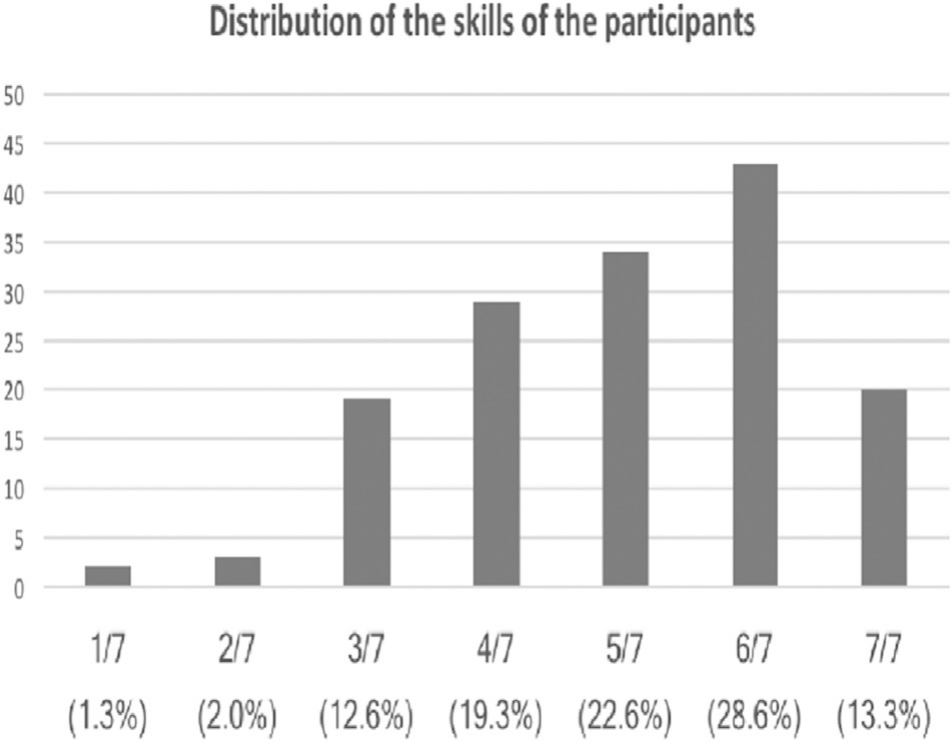
Distribution of the perceived computer skill on a scale from 1 to 7 (in percentage).

The results of the question 4 (*How would you rate your computer skills?*) depicted in Figure 3 underline that the level of knowledge and skill is a very subjective and personal perception. For instance, some accountants have set a very high score (6 out of 7) while some IT professionals have set a smaller level for their own skills. Nevertheless, it is safe to state that whatever the position, most participants consider that they know some things on computers.

Generally, there is no correlation between the perceived computer skill and the position except for the participants who have a very high skill level (7) where 60% of the participants have a technical profile in Information and Communication Technology (developer, IT support, architect, team leader, engineer, etc.). However, we noticed that there is a correlation between the perceived skill and the size of the company. The skill increases slightly with the size of the company as we can see it in the table below.

Now that we have described the results concerning the context and employees, we describe in the next subsection the questions and results that target employees’ awareness, knowledge and perception on security policies and data leakage.

## Feedbacks on Awareness, Knowledge and Perception Toward Security Policies and Data Leakage

In this subsection, we present the results of the questions 5–13 that we have proposed to:

1. Determine if the participants:
  - Use sensitive data (Question 5 and 6);
  - Are aware of any security policies within their companies (Question 7);
  - Have ever been the cause of intentional or unintentional data leakage (Question 8).
2. Collect the feeling and perception of the participants toward security policies and data leak prevention mechanisms.

### Usage of Sensitive Data

Thanks to questions 5 (*In your work, do you have to manage sensitive data?*) and 6 (*If so, do you often manipulate such data?*), we have noticed that 88.7% of participants manipulates sensitive data. We underline that the survey gives a definition of what a sensitive data is, in order to reduce biais induced by participant’s own definition. While filling the form, we present the following definition for sensitive data: “By sensitive data, we mean data containing confidential information that needs to be kept between a restricted set of users (patent, accounting, personal information, etc.).”

Concerning the occurrences, we have seen that 41.1% of participants manage sensitive data frequently (“A significant part of my work is to manipulate sensitive data.”), as against 19.2% who have declared that manipulating such data is rare (“It is never common in my work to manipulate sensitive data.”). Finally, 40% of participants have declared that they manage sensitive data in a quite regular basis (“From time to time, I have to manipulate sensitive data.”).

We can conclude that many employees have to deal with sensitive data (e.g., patent, accounting, personal information) while performing their day to day tasks.

The results obtained showed no correlation between the usage of sensitive data and the size of the company/activity sector or the position of the participants.

### Security Policies Awareness

The next question (i.e., question 7: *In your company, are you aware of the security policies that concern you?*) we have asked to the participants refers to their knowledge regarding security policies within the company. Results shows that 69.5% of them are aware of the security policies and know what they can/cannot do in terms of security (i.e., Access and Transmission Control). However, we underline that roughly a quarter of them (23.8%) have declared that they do not really know what they can/cannot do, despite the fact that they are aware that some security policies exist. Finally, only 6.7% of the participants have declared that they are not aware of any security policies within their company. Based on these results, we can conclude that most participants have some knowledge on the security policies applied within their companies.

The results obtained showed no correlation between awareness regarding security policies and the size of the company/activity sector or the position of the participants.

### Security Policy Bypassing

Another question that we have asked is about bypassing security policies (i.e., question 8: *If you are aware of such policies, have you ever done anything to bypass them?*). Results show that 43.7% of the participants have bypassed a security policy without knowing it, causing a potential unintentional data leakage. Moreover, results show that 35.1% have declared that they have never bypassed a policy, while 21.2% have declared that they have bypassed a policy intentionally.

While exchanging with the 10 participants who gave their email address, we have discovered that some unintentional bypasses have been performed for the following reasons (words in italic are words that have been used by the participants):

- *Lack of awareness*: security policies are not well specified or too obscure for the employees, preventing them to specifically know “what can be done with the document.”
- *Lack of usability*: security policies are blocking an employee on her/his task or context. Thus, she/he has to “improvise.”
- *Carelessness*: the participant does not consider the leakage of a particular data as “critical” or “important.”

The results obtained showed no correlation between the fact to bypass security policies and the size of the company/activity sector or the position/computer skill of the participants. However, concerning, the 65 participants who have answered that they have bypassed the security policies, their response regarding the question 7: “In your company, are you aware of the security policies that concern you?” was as follow:

- “No, and I do not know if they exist” for six of them;
- “Yes, I know what I can/cannot do” for 29 of them;
- “No, I know that they exist but I do not know what I can do/cannot do” for 30 of them.

### Feelings Toward the Lack of Awareness and Knowledge

Based on the previous declarations, we have asked participants their feelings on the lack of awareness and knowledge regarding the security policies of the company. Results of the question 9 (*Do you think that not being aware of such policies is an obstacle for your work?*) show that 61.6% have declared that not knowing the policies is an obstacle for their tasks (while only 17.2% have declared that it is not an obstacle at all). Thus, we can say that the lack of knowledge regarding the security policies can increase the difficulty of performing some tasks, like editing and sending documents such as patent, photos, reports or accounting files.

The results obtained showed no correlation between the feelings toward the lack of awareness and knowledge regarding security policies and the size of the company/activity sector or the position of the participants.

### Data Leakage Perception

In this Section, we present the results of the question 10 *According to you, data leakage can be:*

- A problem for the company (financial loss, image, etc.),
- A problem for the employee (official warning, dismissal, etc.),
- A problem for both,
- A problem for neither of them).

The results show that most of the participants (71.5%) are aware that a data leak can be a very big problem for both companies and employees. Moreover, 23.2% of them have declared that only companies are impacted after a data leakage. Finally, the other choices (i.e., “a problem for the employee” and “a problem for neither of them”) are anecdotal (respectively 4.6 and 0.7%). Thus, we can conclude that most participants are aware that data leakage can be a problem for both companies and employees independently from the size of the company/activity sector and their position.

### Mechanisms to Prevent Data Leakage

Concerning the mechanisms to tackle the problem of data leakage, the results of the question 11 (*Are you aware of security mechanisms implemented within your company?*) show that 55% of participants have declared being aware that their company uses some kind of data leak prevention mechanisms. According to the 13 participants who have commented the form (thanks to the last question, i.e., question 16: *If you have any ideas of mechanisms, some remarks or comments‥*.), we can state that these mechanisms embed network filtering, document ciphering, Document Management System (DMS) and DLP (Symantec). However, no IRM mechanism has been cited yet.

Most of the 13 participants, who have provided comments about the security mechanisms, have a technical profile (i.e., computer skills) and work for large companies in high tech domain (see Tables 5–7).

**TABLE 5:** Number of participants with comments per company size.

Size of the company	Numbers of participants
Micro-enterprise	3 (23%)
SME	2 (15.4%)
MidCaps company	4 (30.8%)
Large company	4 (30.8%)

**TABLE 6:** Number of participants with comments per activity sector.

Activity sector	Numbers of participants
ICT	6 (46.15%)
Industrial	4 (30.8%)
Scientific, research and development	1 (7.7%)
Construction	1 (7.7%)
Insurance/banking/finanicial institution	1 (7.7%)

**TABLE 7:** The level of computer skill of the participants who have provided comments.

Level of computer skill	Numbers of participants
1	0
2	1 (7.7%)
3	0
4	1 (7.7%)
5	3 (23%)
6	5 (38.4%)
7	3 (23%)

Concerning the other results, the survey shows that 21.2% of participants are not aware of security mechanisms within their company (as against 23.8% who have declared that such mechanism exists within their company, without having explicit knowledge of it).

The results obtained showed a correlation between awareness and knowledge regarding data leak prevention mechanisms and the size of the company. This is discussed in “Discussion.”

### Feelings Toward Anti-Data Leakage Mechanisms

Answers gathered thanks to questions 12 (*Do you think that these mechanisms are an obstacle to your work?*) and 13 (*Do you think that these mechanisms are intrusive for employees?*) show interesting results regarding the perception of data leak prevention mechanisms (see Table 8). On one hand, we can underline that for a non-negligible part of participants, these mechanisms are quite blocking. Indeed, if we consider levels 4 and 5, these mechanisms are quite constraining for 44% of participants. However, roughly a quarter of the participants (31.1%) considered that these mechanisms are not very constraining.

**TABLE 8:** Perceived level of constraint on a scale from1 to 5 (5 is very constraining).

Level of constraint	Numbers of participants
1	15/150 (10%)
2	32/150 (21.3%)
3	37/150 (24.6%)
4	51/150 (34%)
5	15/150 (10%)

Concerning the intrusiveness, Table 9 shows that roughly 44% of participants think that these mechanisms are intrusive, while 31% have declared that it is not the case. Thus, we can conclude that the perception of constraint and intrusiveness varies from one individual (and context) to another. However, it is safe to state that these mechanisms are not imperceptible by most employees.

**TABLE 9:** Perceived level of intrusiveness on a scale from 1 to 5 (5 is very intrusive).

Level of intrusiveness	Numbers of participants
1	20/150 (13.3%)
2	25/150 (16.6%)
3	37/150 (24.6%)
4	53/150 (35.3%)
5	15/150 (10%)

The results obtained showed no correlation between the perception and feelings of the participants regarding data leak prevention mechanisms and the size of the company/activity sector or the position of the participants.

## Feedbacks Toward Data Leak Prevention Mechanisms

In this section, we present the results of the questions 14 and 15.

### Preferred Anti-Data Leak Mechanisms

The goal of the question 14 (see below) was to collect information about the preferences of the participants regarding anti-data leak mechanisms in terms of non-intrusiveness, ease-of use and efficiency.

Question 14: According to you, what are the most efficient mechanisms to avoid data leakage?

- Notify users that an action is going to cause a data leakage and let her/him choose (e.g., popup messages).
- Notify users that an action is going to cause a data leakage and prevent this action.
- Send an email to the administrator/manager to notify her/him and automatically prevent the action.
- Send an email to the administrator/manager in order for her/him to choose if the action can be performed or not.
- Automatically deactivate actions that can cause data leakage (for instance, automatically deactivate the “send” button when a confidential attached document is put within an email).
- Prevent the action without notifying the user.
- Other form of mechanisms (you can share ideas at the end of the form).

The obtained results to this question are presented in Table 10.

**TABLE 10:** Proposed mechanisms and their attractiveness.

Proposed mechanisms	Attractiveness (multiple choices)
Notify users that an action is going to cause a data leakage and let her/him choose (ex: popup messages)	68/150 (45.3%)
Notify users that an action is going to cause a data leakage and prevent this action	68/150 (45.3%)
Send an email to the administrator/manager to notify her/him and automatically prevent the action	37/150 (24.6%)
Send an email to the administrator/manager in order for her/him to choose if the action can be performed or not	48/150 (32%)
Automatically deactivate actions that can cause data leakage (for instance, automatically deactivate the “send” button when a confidential attached document is put within an email)	67/150 (44.6%)
Prevent the action without notifying the user	14/150 (9.33%)
Other form of mechanisms	7/150 (4.6%)

Moreover, we underline that an open question (i.e., the last Radio button: *Other form of mechanisms*) has been proposed to allow the participants to cite other mechanisms. Among the obtained results, we can cite:

- Use a Public Key Infrastructure.
- Use different levels of mechanisms depending on the sensitivity and the confidentiality of the data.
- Raise awareness of users (with training, recommendation systems, guidelines, etc.).
- Include a logging mechanism in case of conflict between a user and her/his hierarchy.

As we can see in Table 5, most of the participants would like to be notified that an action is going to cause a data leakage. 45% of the participants prefer to decide to continue or not the action they are performing. 56% of the participants prefer to not take any responsibility and leave the decision to the administrator/manager to prevent or not an action. However, 9% of the participants want to be considered and informed if an action is prevented. 69% are in favor of automatic actions (i.e., automatically prevent/deactivate action).

### Security vs “Freedom”

The goal of the question 15 was to ask the participants if they would prefer a mechanism that let them decide, but might let them perform a data leakage (more “freedom”), or if they would prefer a mechanism that prevent them to perform unintentional data leakage, without letting them decide (more security). Results show that more than 55% of them prefer security over freedom, whatever the type of participants (i.e., positions and skills). However, we have discovered interesting correlation with the size of the companies. These correlations are presented in the next subsection.

## Discussion

In this final subsection, we discuss correlations we have identified between answers in order to underline some interesting results regarding the link between the size of a company and perception toward security. These correlations are presented in the next subsections.

### Correlation Between the Size of the Company and Security vs. Freedom

By comparing the size of the companies and the previous question (i.e., question 15: *In the end, you would prefer a mechanism that let you decide, but might let you perform data leakage or a mechanism that prevent you from unintentional data leakage, but does not let you decide*), we have discovered that participants from very small companies tend to prefer a mechanism that promotes security over a certain freedom.

Indeed, Table 11 shows that, in the case of middle-sized, big, and very big companies, the ratio between answers are quite equivalent. In the case of very small companies, the distribution is very different, underlining that employees of these companies rather prefer a mechanism that prevent them to leak data, even if this mechanism is too restrictive. In order to have a better understanding of these results, we have asked some participants, in small companies, to explain their choice. Among the answers, we can highlight the following remarks (the words in italic are the ones used by the participants):

- “The data are my bread and butter, I cannot lose them over a mistake.” (Entrepreneur).
- “It can be a big problem for me to send my pictures to the wrong person.” (Professional photograph).
- “As a member of a small company, everyone knows everyone, I guess it would be a shame to leak a personal information.” (Secretary).

**TABLE 11:** Correlation between the perception of mechanisms and the size of the companies.

Company size	Number of participants	Security over “freedom”	“Freedom” over security
Micro-enterprise	46/150 (30.6%)	(73.9%)	(26.1%)
SME	32/150 (21.3%)	(53.1%)	(46.9%)
MidCaps company	28/150 (16.6%)	(57.2%)	(42.8%)
Large company	44/150 (29.3%)	(47.7%)	(52.3%)

These answers show that a smaller infrastructure tends to make the leaker “more responsible,” probably because of the social proximity with the other employees.

As a conclusion, we can say that the smaller the company, the bigger the need for security. Employees of bigger companies prefer having more freedom over security.

### Correlation Between the Size of the Company and Awareness Toward the Security Mechanisms

When analyzing the results obtained for the question 11 (Are you aware of security mechanisms* implemented within your company?), we have noticed that there is a correlation between awareness of the participants toward the security mechanisms and the size of the company (see Table 12 **)**. Indeed, participants from bigger infrastructures tend to have a better awareness and knowledge of the security mechanisms used by their company. We hypothesize that bigger infrastructures are more likely to inform, raise the awareness and educate the employees, thanks to dedicated guidelines and training.

**TABLE 12:** Correlation between the awareness of security mechanisms and the size of the company.

Company size	Number of participants	Yes	No, (but know they exist)	No, (do not know if they exist)
Micro-enterprise	46/150 (30.6%)	(58.7%)	(21.7%)	(19.6%)
SME	32/150 (21.3%)	(46.9%)	(50%)	(3.1%)
MidCaps company	28/150 (16.6%)	(75%)	(25%)	0
Large company	44/150 (29.3%)	(93.2%)	(6.8%)	0

## Conclusion and Future Works

In this article, we have presented the results of an online survey that we have proposed to 150 employees from different companies. Among other things, the survey has been able to gather information on the context (e.g., size of the company, sector of activity) and the participants themselves (e.g., computer’s skill, position). The survey has been used to have insights on the employee’s knowledge and perception toward sensitive data and security policies. However, unlike existing works, we have been able to gather information on the attitude and perception of the employees regarding data leakage within companies and the used prevention mechanisms.

The results have shown that the mechanisms used by the companies are quite known by end-users. However, many of them considered that these mechanisms are quite intrusive and blocking for their work. In addition, the survey has underlined that most of the participants have been involved in both intentional and unintentional data leakage for several reasons, including lack of awareness, lack of usability and carelessness. Moreover, we have spotted out differences between small and big companies. Indeed, the results have shown that employees of small infrastructures tends to prefer mechanisms that prevent data leak, even if this protection does not let them decide what to do. Concerning the awareness, the results also show that employees of bigger companies tend to be more aware and trained when it comes to security.

Finally, we have used the survey to ask participants the mechanisms they would prefer in order to prevent them from unintentional data leakage. Based on the collected results, we will integrate the favorite solutions to an existing data leakage prevention policy engine. Moreover, we aim at using the context (i.e., type and sensitivity of the data, company’s guideline, etc.) and the user preferences to dynamically change these mechanisms in order to provide contextual, usable, acceptable, non-intrusive and user-friendly anti-data leakage mechanisms.

## Data Availability Statement

The raw data supporting the conclusions of this article will be made available by the authors, without undue reservation.

## Ethics Statement

Ethical review and approval was not required for the study on human participants in accordance with the local legislation and institutional requirements. Written informed consent for participation was not required for this study in accordance with the national legislation and the institutional requirements.

## Author Contributions

YB and KB designed the study. YB conducted the study and analyzed the results. KB and MR supervised the work done by YB. YB and KB contributed to manuscript revision. All the authors approved the submitted version.

## Funding

This work was supported partly by the FUI (Fond Unique Interministriel) project named 4TRAX.

## Conflict of Interest

The authors declare that the research was conducted in the absence of any commercial or financial relationships that could be construed as a potential conflict of interest.

## Footnotes

b
http://www.zdnet.com/article/trend-micro-makes-dlp-move-symantec-stands-pat/.

c
https://www.forcepoint.com/fr/product/web-filtering/websense-web-filter-security

d
http://www.trendmicro.fr/grandes-entreprises/protection-des-donnees/prevention-des-pertes-de-donnees-integree/

e
https://www.rsa.com/en-us

f
https://www.symantec.com/fr/fr/data-loss-prevention/

g
http://palisadesystems.com/

h (http://www.seclore.com/).

i
https://technet.microsoft.com/en-us/library/dd638140(v=exchg.150).aspx

j
http://www.covertix.com/

k
http://france.emc.com/enterprise-content-management/information-rights-management.htm.

l
https://www.cryptzone.com/pdfs/Whitepapers/CryptzoneNetwork-Access-Security-Survey-2015

m
http://www.sans.org/readingroom/whitepapers/analyst/cybersecurity-professionaltrends-survey-34615

n
https://www.clearswift.com/solutions/adaptive-data-loss-prevention

o
https://www.teramind.co/product/dlp-data-loss-prevention

p
https://goo.gl/forms/4HcEFZ8ifNWXRPM52

q
https://goo.gl/forms/TknBWkGnsFySgryT2

## References

[B1] Alawneh M. Abbadi I. M. (2008). “Preventing information leakage between collaborating organisations,” in Proceedings of the 10th international conference on electronic commerce Innsbruck, Austria, August 2008 (New York, NY: ACM).

[B2] Alotaibi M. Furnell S. Clarke N. (2016). “Information security policies: a review of challenges and influencing factors,” in The 2016 11th international conference for internet technology and secured transactions (ICITST) Barcelona, Spain, 352–358.

[B3] Bai W. Kim D. Namara M. Qian Y. Kelley P. G. Mazurek M. L. (2016). An inconvenient trust: user attitudes toward security and usability tradeoffs for key-directory encryption systems, in Proceedings of the 12th Symposium On Usable Privacy and Security (SOUPS 2016), Denver, CO, June 22–24, 2016.

[B4] Banerjee A. Naumann D. A. (2004). “History-based access control and secure information flow,” in International workshop on construction and analysis of safe, secure, and interoperable smart devices (New York, NY: Springer), 27–48.

[B5] Beautement A. Becker I. Parkin S. Krol K. Sasse A. (2016). “Productive security: a scalable methodology for analysing employee security behaviours,” in Symposium on usable privacy and security (SOUPS), Denver, CO, June 22–24, 2016, 253–270.

[B6] Bell D. E. LaPadula L. J. (1973). Secure computer systems: mathematical foundations. ESD/AFSC Technical Rep No. ESD-TR-73-278, DTIC Document.

[B7] Biba K. J. (1977). Integrity considerations for secure computer systems. Technical report, DTIC Document.

[B8] Caputo D. Maloof M. Stephens G. (2009). Detecting insider theft of trade secrets. IEEE Secur. Priv. 7 (6), 14–21. 10.1109/msp.2009.110

[B46] Cisco Systems (2009). Data leakage worldwide: Common risks and mistakes employees make. Cisco Systems, Inc. Available at: http://www.cisco.com/en/US/solutions/collateral/ns170/ns896/ns895/white_paper_c11-499060.html.

[B9] Corrad A. Montanari R. Tibaldi D. (2004). “Context-based access control management in ubiquitous environments,” in Proceedings of the third IEEE international symposium on network computing and applications, Cambridge, MA, 2004 (New York, NY: IEEE), 253–260.

[B10] Chae C.-J. Shin Y. Choi K. Kim K.-B. Choi K.-N.. (2015). A privacy data leakage prevention method in p2p networks. Peer-to-Peer Netw. Appl. 9 (3), 508–519. 10.1007/s12083-015-0371-x

[B11] di Vimercati S. D. C. Foresti S. Paraboschi S. Pelosi G. Samarati P. (2011). “Efficient and private access to outsourced data,” in 31st International conference on distributed computing systems (ICDCS) Minneapolis, MN, June 20–24, 2011 (New York, NY: IEEE), 710–719.

[B12] Felt A. P. Reeder R. W. Ainslie A. Harris H. Walker M. Thompson C. (2016). “Rethinking connection security indicators.” in Proceedings of the 12th Symposium On Usable Privacy and Security (SOUPS 2016), Denver, CO, June 22–24, 2016, 1-14

[B13] Gafny M. A. Shabtai A. Rokach L. Elovici Y.. (2010). “Detecting data misuse by applying context-based data linkage,” in Proceedings of the 2010 ACM workshop on Insider threats Chicago, IL, October 2010 (New York, NY: ACM), 3–12,

[B14] Harel A. Shabtai A. Rokach L. Elovici Y. (2010). “M-score: estimating the potential damage of data leakage incident by assigning misuseability weight,” in Proceedings of the 2010 ACM workshop on Insider threats, IL, October 2010 (New York, NY: ACM), 13–20.

[B15] Harel A. Shabtai A. Rokach L. Elovici Y. (2012). M-score: a misuseability weight measure. IEEE Trans. Depend. Secure Comput. 9 (3), 414–428. 10.1109/tdsc.2012.17

[B16] Harrison M. A. Ruzzo W. L. Ullman J. D.. (1976). Protection in operating systems. Commun. ACM. 19 (8), 461–471. 10.1145/360303.360333

[B17] Hu V. C. Ferraiolo D. Kuhn R. Friedman A. R. Lang A. J. Cogdell M. M. (2013). Guide to attribute based access control (abac) definition and considerations (draft). NIST Spec. Publ. 800, 162.

[B18] Kagal L. Finin T. Joshi A. (2001). Trust-based security in pervasive computing environments. Computer 34 (12), 154–157. 10.1109/2.970591

[B19] Kandala S. Sandhu R. Bhamidipati V. (2011). “An attribute based framework for risk-adaptive access control models,” in 2011 sixth international conference on availability, reliability and security (ARES) Vienna, Austria, August 22–26, 2011 (New York, NY: IEEE), 236–241.

[B20] Karp A. Haury H. Davis M. (2010). “From abac to ZBAC: the evolution of access control models. ISSA J. 8. 22-30

[B21] Kirlappos I. Parkin S. Angela Sasse M. (2014). “Learning from “Shadow Security”: why understanding non-compliant behaviors provides the basis for effective security,” in Proceedings of Workshop on usable security San Diego, CA, February 23, 2014. 10.14722/usec.2014.23007

[B45] Ko R. K. L. Tan A. Y. S. Gao T. (2014). “A Mantrap-Inspired, User-Centric Data Leakage Prevention (DLP) Approach,” in 6th International Conference on Cloud Computing Technology and Science, Singapore, 1033-1039. 10.1109/CloudCom.2014.23

[B22] Kumaraguru P. Cranor L. F. (2005). Privacy indexes: a survey of westin’s studies. Technical report. Carnegie Mellon University, School of Computer Science, Institute for Software Research International.

[B23] Lampson B. W. (1974). Protection. ACM SIGOPS. Oper. Syst. Rev. 8 (1), 18–24. 10.1145/775265.775268

[B24] Li H. Peng Z. Feng X. Ma H. (2015). “Leakage prevention method for unstructured data based on classification,” in Proceedings of the 6th International Conference on Applications and techniques in information security Beijing, China, November 4–6, 2015 (New York, NY: Springer), 337–343.

[B25] Liu B. Andersen M. S. Schaub F. Almuhimedi H. Zhang S. Sadeh N. (2016). “Follow my recommendations: a personalized privacy assistant for mobile app permissions,” in Proceedings of 12th Symposium on usable privacy and security Denver, CO, June 22-24, 2016.

[B26] Mathew S. Petropoulos M. Ngo H. Q. Upadhyaya S. (2010). “A data-centric approach to insider attack detection in database systems,” in International workshop on recent advances in intrusion detection Ottawa, ON, Canada, September 15–17, 2010 (New York, NY: Springer), 382–401. 10.1007/978-3-642-15512-3_20

[B27] Pahnila S. Siponen M. Mahmood A (2007). “Employees’ behavior towards IS security policy compliance,” in Annual Hawaii international conference on system sciences (HICSS) Waikoloa, HI, January 3–6, 2007 (New York, NY: IEEE), 156b.

[B28] Rhee H.-S. Kim C. Ryu Y. U. (2009). Self-efficacy in information security: its influence on end users’ information security practice behavior. Comput. Secur. 28 (8), 816–826. 10.1016/j.cose.2009.05.008

[B29] Rosenblatt W. Mooney S. Trippe W. (2001). Digital rights management: business and technology. Hoboken: John Wiley & Sons.

[B30] Ruoti S. Neill M. O. Zappala D. Seamons K. (2016). “User attitudes toward the inspection of encrypted traffic,” in Proceedings of SOUPS 2016: twelfth symposium on usable privacy and security, Denver, CO, June 22–24, 2016, 131–146.

[B31] Saltzer J. H. Schroeder M. D. (1975). The protection of information in computer systems. Proc. IEEE. 63 (9), 1278–1308. 10.1109/proc.1975.9939

[B32] Sandhu R. S. Coyne E. J. Feinstein H. L. Youman C. E. (1996). Role-based access control models. Computer 29 (2), 38–47. 10.1109/2.485845

[B33] Shabtai A. Elovici Y. Rokach L. (2012). A survey of data leakage detection and prevention solutions. Berlin: Springer Science & Business Media).

[B34] Stobert E. Biddle R. (2014). “The password life cycle: user behaviour in managing passwords,” in Proceedings of the SOUPS. 10.1145/2683467.2683471

[B35] Soliman A. H. Ibrahim M. H. El-Ramly S. H. (2015). “Enhancing efficiency of enterprise digital rights management,” in 2015 International conference on advanced computer science and information systems (ICACSIS) Denton, TX, July 2015 (New York, NY: IEEE), 91–96.

[B36] Subramanya S. R. Yi B. K. (2006). Digital rights management. IEEE Potent. 25 (2), 31–34. 10.1109/mp.2006.1649008

[B37] van Beek M. (2007). Comparison of enterprise digital rights management systems. Master Thesis Computer Science MT Advice report. Aia Software. Thesis number 565. June 22, 2007, Radboud University Nijmegen.

[B38] Van Tassel J. (2006). Digital rights management. London: Taylor & Francis.

[B39] Wan Ismail W. B. Yusof M. (2018). Mitigation strategies for unintentional insider threats on information leaks. Int. J. Secur. Appl. 12 (1), 37–46. 10.14257/ijsia.2018.12.1.03

[B41] Workman M. Bommer W. H. Straub D. (2008). Security lapses and the omission of information security measures: a threat control model and empirical test. Comput. Hum. Behav. 24(6), 2799–2816. 10.1016/j.chb.2008.04.005

[B42] Yang J.-H. Sun H.-M. Chen P.-L. (2013). An enterprise digital right management scheme with anonymous trust for mobile devices. Informatica 37 (3), 307–313.

[B43] Yu Y. Chiueh T.-C. (2004). Enterprise digital rights management: solutions against information theft by insiders. Research Proficiency Examination (RPE) report TR-169, Department of Computer Science, Stony Brook University, 33.

[B44] Zilberman P. Dolev S. Katz G. Elovici Y. Shabtai A. (2011). “Analyzing group communication for preventing data leakage via email,” in 2011 IEEE international conference on intelligence and security informatics (ISI) Beijing, China, August 2011 (New York, NY: IEEE), 37–41.

